# Association of Chronic *Toxoplasma gondii* Infection with Pro-Inflamatory Cytokine Interleukin (IL)-12 Responses in Type-2 Diabetes Mellitus Patients of Bangladesh

**DOI:** 10.1155/2023/3885160

**Published:** 2023-05-08

**Authors:** Tamanna Ashraf, Pankaj Kumar Sarker, Md. Ismail Hosen, Atiqur Rahman, A. K. M. Mahbub Hasan, Taibur Rahman

**Affiliations:** ^1^Laboratory of Infection Biology, Department of Biochemistry and Molecular Biology, University of Dhaka, Dhaka 1000, Bangladesh; ^2^Laboratory of Biochemistry, Dinajpur Diabetic Hospital, Dinajpur, Bangladesh; ^3^Laboratory of Clinical Biochemistry and Translational Medicine, Department of Biochemistry and Molecular Biology, University of Dhaka, Dhaka 1000, Bangladesh

## Abstract

*Toxoplasma gondii* is an intracellular protozoan parasite that causes toxoplasmosis in around one-third of the world population, particularly in pregnant women and immunocompromised individuals. Diabetes mellitus (DM) is one of the most severe global health challenges in the 21st century, and especially, type-2 diabetes mellitus (T2DM) accounts for 90% of the diabetes cases diagnosed globally. In Bangladesh, the rate of T2DM is rising gradually with the improvement in living standards. The aim of this study is to find out the correlation between latent toxoplasmosis and T2DM, emphasizing the pro-inflammatory cytokine immunity. For this, 100 (*N* = 100) patients with T2DM and 100 (*N* = 100) healthy controls were enrolled to determine the seroprevalence of toxoplasmosis using enzyme-linked immunosorbent assay (ELISA). In addition, ELISA was also performed to determine the level of pro-inflammatory cytokine, interleukin (IL)-12, to understand its role in the development of toxoplasmosis. In our study, 39.39% of the T2DM patients were positive with anti-*T. gondii* Immunoglobulin G by ELISA, whereas the rate of seropositivity in healthy controls was 39.73%. We did not find significant association between *T. gondii* infection and T2DM, but our data confirmed a high prevalence of chronic toxoplasmosis in Bangladeshi population. From hematology tests, it was found that the T2DM patients had significantly lower levels of total white blood cells (*P* = 0.0015), circulating eosinophils (*P* = 0.0026), and neutrophils (*P* = 0.0128) than the healthy controls. On the other hand, the levels of lymphocytes (*P* = 0.0204) and monocytes (*P* = 0.0067) were significantly higher in patients. Furthermore, *T. gondii* infected T2DM patients had significantly higher levels of IL-12 than the healthy controls (*P* = 0.026), suggesting a link between parasitic infection and IL-12 secretion. Further studies are to be performed to find out the exact cause of high prevalence of chronic *T. gondii* infection in Bangladeshi population.

## 1. Introduction

Toxoplasmosis is a disease caused by the protozoan parasite, *Toxoplasma gondii.* It is a globally widespread parasitic infection having varying seroprevalence rates based on geographical locations. In the United States and the United Kingdom, the infection rate is estimated to be 16–40% of the total population, while it is 50–80% in Central and South America, and in Europe, respectively [1]. The symptoms of the disease are rarely noticeable in most cases though it can exhibit manifestations like fever, sore throat, headache, fatigue, and muscle pain. Mostly, immunocompromised individuals and pregnant women are the worst sufferers of the clinical complications of chronic toxoplasmosis. During pregnancy, the mother can contribute to the congenital *T. gondii* infection leading to severe damaging occurrences in the fetus, such as intra-cerebral calcifications, retinochoroiditis, etc., which ultimately leads to diminished mental development as well as blindness [2]. Immunocompromised individuals such as cancer patients, AIDS patients, and organ-transplant patients are critically vulnerable to *T. gondii* infection. The complications incurred by these groups include encephalitis, convulsions, hemiparesis, coma, and reflex changes [1].

In modern times, the occurrence of diabetes mellitus, a disorder in the metabolic system, is increasing rapidly. It is characterized by high blood sugar level as a result of either insufficient secretion of insulin by the pancreas or by becoming insulin resistance where the body is not responding to insulin. This insulin resistance is observed in type-2 diabetes mellitus (T2DM), which is also called as non-insulin dependent diabetes mellitus (NIDDM). Poor insulin response contributes to the occurrence of co-morbid situations associated with abnormal glycemic control in diabetes [3]. Different studies reported that the susceptibility to various kinds of infections, i.e., *Helicobacter pylori,* is found to be positively correlated with higher HbA1C levels resulting from diminished insulin secretion and high body mass index (BMI) scores [4]. The higher vulnerability to infections in diabetes mellitus patients may result from the decline in the immune response, which may include impairments in the immune cell counts [5].

Cytokines are the protein molecules mainly involved in regulating immune responses during infection. Different cytokines are secreted from two distinct groups of T lymphocytes, especially by helper T cells (Th cells). Two subsets of Th cells, Th1 and Th2, have their separate cytokine profiles, counterbalancing each other. To be clearer, Th1 cells are responsible for the production of the pro-inflammatory cytokines, for instance, interleukin (IL)-12 (IL-2), Tumor necrosis factor-*α*, and Interferon-*γ* (IFN-*γ*), whereas Th2 cells produce the anti-inflammatory cytokines, such as IL-4, IL-10, and IL-5 [6]. Studies have suggested an impairment in the balance between Th1 and Th2 profiles during diabetes mellitus, which may lead to dysregulated immunity against pathogen [3].

The pro-inflammatory cytokine, IL-12, acts as a bridge between the innate and adaptive immune responses. It is produced by antigen-presenting cells (APC) and different phagocytic cells, including neutrophils, natural killer cells, dendritic cells, and macrophages. IL-12 plays a crucial role in the proliferation of T lymphocytes and Natural Killer (NK) cells mediating the cytotoxicity by immune cells [7]. IL-12 also helps in the secretion of IFN-*γ* (a pro-inflammatory cytokine) by T-cells, APCs, and the NK cells. The effect of the initiation of IFN-*γ* signaling on immune system inhibits Th2 cell differentiation. This ultimately activates the macrophages and cytotoxic T (Tc) cells for enhanced phagocytic activity and production of free radical nitric oxide (NO). All these mechanisms ultimately support the reduction of parasitic load in the host during infection [8]. The objective of this study was to investigate the rate of seroprevalence of *T. gondii* infection in T2DM patients and to determine the effect of the parasitic infection on pro-inflammatory cytokine IL-12 responses in T2DM patients.

## 2. Method and Materials

### 2.1. Study Design and Sample Collection

T2DM patients were selected as the target population for this study. A total of 100 (*N* = 100) T2DM patients from the “Heath Care Development Project” at the Dinajpur Diabetes O Swasthoseba Hospital, Dinajpur, Bangladesh, were enrolled in this study. In addition, 100 (*N* = 100) healthy subjects were included from the same region as controls. Along with a structured questionnaire, all data regarding the fasting blood glucose level and the level after 2 hours drinking of 75 g of glucose solution as well as the complete blood count (CBC) reports of the patients were collected.

The inclusion criteria for the patients were that they had to be diagnosed with T2DM with fasting plasma glucose level>6.9 mmol/L and HbA1C level>6.5%. The age limit of the study participants was 30 years and above. Only two cases with ages under 30 years were included following the confirmation of T2DM. In addition to the diagnosis of diabetes, the patients had to have CBC test performed. Patients with various forms of diabetes other than T2DM or with other severe illnesses were excluded from the study. Pregnant women and children were not included in the study. BMI was recorded from height and weight and was classified as underweight (<18.5 kg/m^2^), normal weight (18.5–24.9), overweight (25–29.9), and obese (30 or more).

### 2.2. Serum Separation

A total of 5 mL of blood was collected from each study subject. Then, serum was separated from the blood samples by centrifugation of whole blood at 2000×*g* for 15 minutes immediately after collection. Following centrifugation, 1.0 mL of the supernatant designated as serum was transferred into a 1.5 mL microcentrifuge tube and was stored at −20°C for experimental analysis.

### 2.3. Determination of Anti-*T. gondii* IgG by Enzyme-Linked Immunosorbent Assay

The presence of anti-*T. gondii* Immunoglobulin G (IgG) in serum of the patients and the healthy controls was measured by enzyme-linked immunosorbent assay (ELISA) [Boster Human Toxoplasma IgG ELISA Kit (Boster BioTech, CA, USA)] according to manufacturer's guidelines. The antibody index for each sample was determined and compared with the cut-off value for interpretation.

### 2.4. Determination of the Concentration of IL-12 by ELISA

ELISA was performed in the serum to determine the concentrations of the cytokine IL-12 in T2DM patients and the healthy controls using Boster Picokine™ Human IL12A Pre-Coated ELISA kits as per manufacturer's instruction. The concentration was then determined by interpolating the absorbance value of the samples on the standard curve. Briefly, 100 *μ*L of the standard, samples, or the controls was added into each well, and 100 *μ*L of the sample diluent was added into the blank well. After covering the strips with the plate sealer, the plate was incubated at room temperature for 120 minutes. Following incubation, the sealer was kept aside, and the liquid inside was discarded into a waste container. The plate was inversely put onto a paper towel to gently blot any remaining liquid. Without letting the wells to completely dry at any point, 100 *μ*L of the prepared 1× Biotinylated Anti-Human IL12A antibody was added to each well. The plate was covered with the sealer and again incubated at room temperature for 90 minutes. After that, the liquid was discarded, and the plate was blotted onto a paper towel and the wells were washed with 300 *μ*L of 1× wash buffer three times. The wash buffer was then discarded into the waste container, the plate was gently blotted, and 100 *μ*L of the prepared 1× avidin–biotin–peroxidase complex was added into each well. A plate sealer was used to cover the plate, and again, the plate was incubated for 40 minutes at room temperature. Subsequently, the liquid in the wells was discarded, and the plate was washed five times with the 1× buffer. After discarding the buffer and gently blotting the plate, 90 *μ*L of the color developing reagent was added to each well. The plate was covered with a plate sealer and incubated in dark for 30 minutes at room temperature. Lastly, 100 *μ*L of the Stop solution was added to each well. The color changed immediately to yellow. The absorbence was measured within 30 minutes of stopping the reaction with a microplate reader at 450 nm.

### 2.5. Statistical Analysis

Data were analyzed in the GraphPad Prism 8.0.2 software. To compare categorical data, chi-square test was performed. Welch's *t*-test was performed for normally distributed variables; otherwise, Mann–Whitney test was performed. Spearman's correlation test was performed in the variables to prepare a correlation matrix. *P* value of <0.05 was considered to be statistically significant.

## 3. Results

In this study, the average age of the T2DM patients was 45.75 ± 11.23 (mean ± SD) years, where 58% of them were female. The fasting plasma glucose of these patients was 11.85 ± 3.75 mmol/L (mean ± SD). Sixty-two (62%) of the patients had BMI in the normal range, 20% were overweight, 12% were obese, and 6% were underweight. The summary of the demographical data of the patients is shown in Table 1.

**Figure 1:**
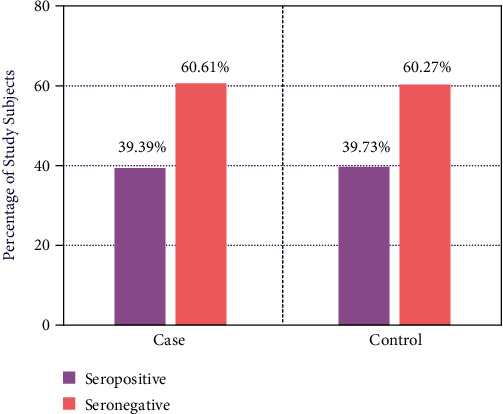
The rate of seropositivity of *T. gondii* infection in both T2DM cases (*n* = 99) and healthy controls (*n* = 99) was measured by ELISA. One sample from each group was excluded due to insufficient data. The rate of seropositivity of *T. gondii* in T2DM patients was approximately similar to healthy controls. The percentage of anti-*T. gondii* IgG positive T2DM cases and healthy controls was calculated and presented. *P*-value was calculated using chi-square test between healthy controls and T2DM cases and considered significant if *P* < 0.05. There were no statistically significant differences between T2DM cases and healthy controls.

**Figure 2:**
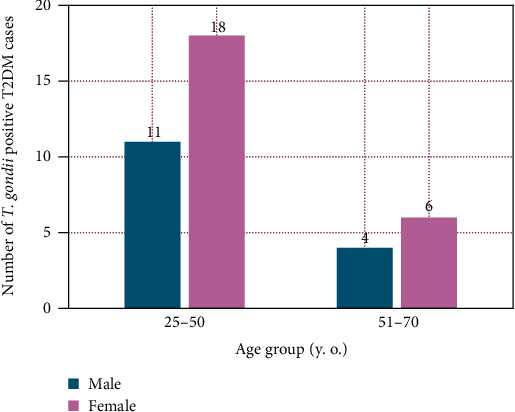
Comparison of seropositivity of *T. gondii* in T2DM patients with age groups and genders. The anti-*T. gondii* IgG positive and negative cases for age groups 25–50 and 51–70 years were calculated and expressed as the absolute number. In addition, the anti-*T. gondii* IgG positive and negative cases were compared for both male and female groups and expressed as the absolute number.

The rate of prevalence of *T. gondii* infection in T2DM patients and healthy subjects was measured by ELISA. However, approximately 39.39% of the case group were seropositive with anti-*Toxoplasma* IgG antibody, whereas the rate of seropositivity was 39.73% in healthy control group (Figure 1). Our data showed that the rate of chronic *T. gondii* infection was higher in both cases and healthy controls, but the differences were not statistically significant. The majority of the *T. gondii* infected T2DM patients were within the age range of 25–50 years old. A total of 29 patients from the 39 infected cases were categorized in the mentioned age group. The female population was found to be more prone to be infected with the parasite (Figure 2).

**Figure 3:**
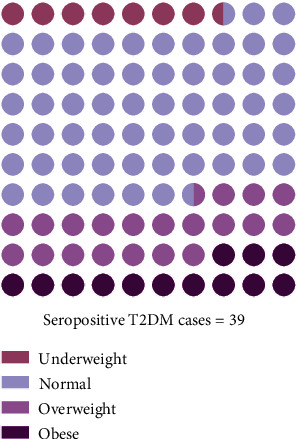
*T. gondii* infection prevalence in four BMI categories among the seropositive patients. 58.97% of the population was normal weight, 20.51% was overweight, 12.82% was obese, and, lastly, 7.69% was underweight patients.

Categorizing the seropositive patients into four different BMI groups, the rate of the prevalence of the infection in those groups was observed. *T. gondii* infection rate was highest in the normal weighted patients (58.97%). The underweight, overweight, and obese groups exhibited 7.69%, 20.51%, and 12.82% of *T. gondii* infection rates, respectively (Figure 3).

**Figure 4:**
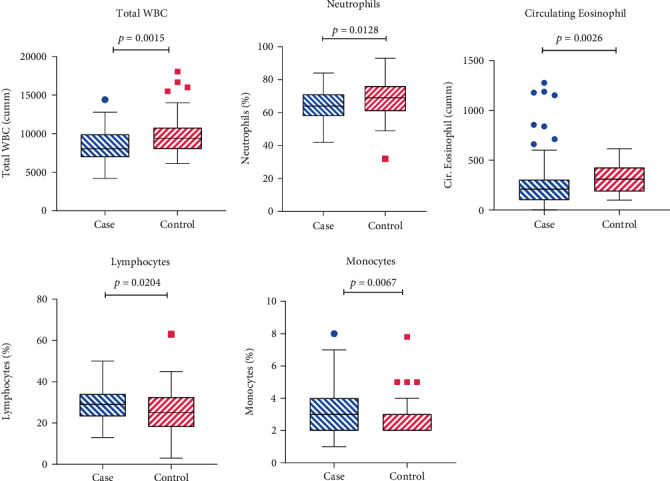
The blood cell counts were compared between the case and control groups. The levels of total WBC, neutrophils, and circulating eosinophils were significantly lower in the case group than in control group (*P* = 0.0015, *P* = 0.0128, and *P* = 0.0026). The levels of lymphocytes and monocytes were significantly reduced in the case group than in control group (*P* = 0.0204, *P* = 0.0067).

The clinical indices acquired from the hematology test reports of the T2DM patients and the healthy controls were compared and summarized in Supplementary Table S1. There were considerable alterations in the blood cell counts between those two groups. In our study, the mean ± SD of total white blood cells (WBC) count in T2DM patients and the healthy controls was 8347 ± 2046 cumm and 9833 ± 2621 cumm, respectively, exhibiting a significant decrease in the case group (*P* = 0.0015). Mean ± SD of neutrophil level in the T2DM patients was 63.86 ± 9.108%, which was 68.65 ± 12.01% in the healthy controls. The levels of neutrophils and circulating eosinophils were also significantly lower in the diabetes patients than the healthy controls (*P* = 0.0128, *P* = 0.0026). On the contrary, the study indicated a significant increase in the levels of lymphocytes and monocytes in the type-2 mellitus patients than healthy controls (*P* = 0.0204, *P* = 0.0067). In the diabetes patients, the mean ± SD of lymphocytes and monocytes levels was 29.50 ± 8.320% and 3.380 ± 1.462%, respectively, whereas in the control group, the mean levels of lymphocytes and monocytes were 25.29 ± 11.45% and 2.809 ± 1.109% (Figure 4).

Correlation analysis performed in the variables present in the case group data is presented in Supplementary Figure S1, showing a positive correlation between fasting plasma glucose (FPG) and post-prandial plasma glucose (PPG), total WBC, and neutrophils. A strong negative correlation has been exhibited between lymphocytes and neutrophils; neutrophils and circulating eosinophils; and total WBC and lymphocytes.

IL-12 concentration varied between the case and control groups, both *T. gondii* infected and uninfected. The mean ± SEM of the concentrations of IL-12 cytokine in infected and uninfected T2DM patients was 9.792 ± 2.686 pg/mL and 5.533 ± 2.082 pg/mL, respectively.

**Figure 5:**
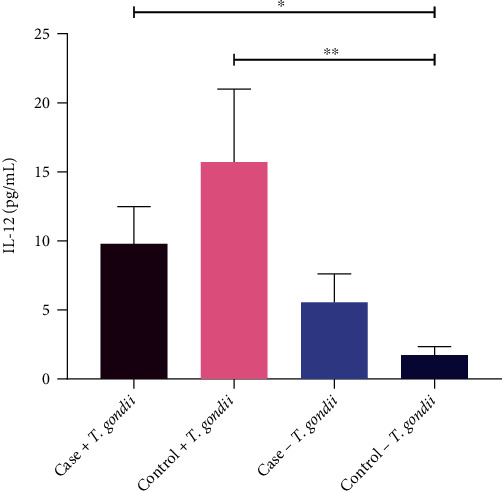
Level of interleukin (IL)-12 in serum of *T. gondii* positive T2DM patients and healthy controls. The study subjects were examined as *T. gondii* positive T2DM patients, *T. gondii* positive healthy controls, *T. gondii* negative T2DM patients, and healthy controls as well. Concentration (pg/mL) of IL-12 was measured through enzyme-linked immunosorbent assay (ELISA) in serum of the above-mentioned groups. Here, *X*-axis represents sample population groups, and *Y*-axis represents the concentration of IL-12 in picogram per milliliter (pg/mL). Data were expressed as mean ± SEM (standard error mean). Significant differences were calculated by unpaired students *t*-test and considered significant if *P* < 0.05.

The mean concentrations of IL-12 in infected and uninfected controls were 15.70 ± 5.290 pg/mL and 1.725 ± 0.6037 pg/mL, respectively. There was a significant increase in the IL-12 concentration in the infected patients (*P* = 0.026) and the infected healthy controls (*P* = 0.004) than the uninfected controls (Figure 5).

**Figure 6:**
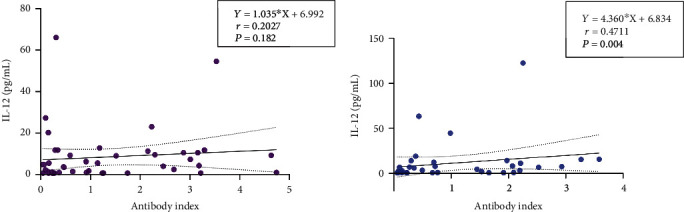
Correlation analysis between antibody index and IL-12 concentrations. The *X*-axis shows the antibody index, and *Y*-axis represents the concentration of Il-12 (pg/mL). GraphPad Prism Version 9.0 was used to prepare the figure. A positive correlation of IL-12 level and antibody index was observed in the control group.

Correlation analysis was performed between the data of antibody index obtained from ELISA and the concentration of IL-12 in both the case and control groups. The result of the analysis in the control group displayed a significant positive correlation (*P* = 0.004) between these variables (Figure 6).

Including IL-12 and antibody index in the dataset, a correlation analysis was performed in the case group, and the result is summarized in Supplementary Figure S2, which indicates a significant positive correlation between FPG and PPG, neutrophils and total WBC, and antibody index and BMI. A significant negative correlation is observed between neutrophils and circulating eosinophils, lymphocytes and total WBC, lymphocytes and neutrophils, and, lastly, monocytes and lymphocytes.

## 4. Discussion

*T. gondii* infection is counted as one of the “Five Neglected Parasitic Infections” by the Centers for Disease Control [9]. The rate of this infection and the vulnerability to severe cases of the disease in humans largely depends on the strain availability in a certain geographical location as well as the host's immune responses to the parasite. Several studies on the function of immune cells in T2DM patients reported that the occurrence of T2DM is followed by dysregulated immune cell activation [10]. T2DM patients were also reported to be more affected by various infections [11]. The phenomenon of T2DM in Bangladeshi population is not a rare case in the modern days because of the inheritance of western lifestyle and physical inactivity; hence, the number of patients continues to increase at an alarming rate. This study was conducted to address the possible association of *T. gondii* infection with T2DM in Bangladeshi population as well as to outline the immune response in regard to cytokine secretion during the parasitic infection.

In this study, we explored the rate of seroprevalence of *T. gondii* infection in T2DM patients and compared it with that of the healthy controls using ELISA. We found 39.39% T2DM patients were positive for chronic toxoplasmosis characterized by the presence of anti-*T. gondii* IgG antibodies in their serum, whereas 39.73% of healthy subjects were positive for anti-*T. gondii* IgG antibodies.

The high prevalence of toxoplasmosis in the sample population could not be concluded as an association between toxoplasmosis and T2DM in our study, as the rate of prevalence of the infection did not differ significantly between T2DM patients (39.39%) and healthy controls (39.73%). Similar results were observed in the studies on Mexican, Australian, and American populations [9, 12, 13]. Strain differences and geographical bias may lead to altering results from the studies conducted in Middle-East countries [14–16].

The prevalence of the infection was higher in the female population of childbearing age, which is 25–50 years old group. The reason behind this may include household chores, which demands frequent contact with possibly contaminated soil, water, vegetable, meat, and even infected cat. This is a very risky circumstance for public health as it has the potential to transmit the *T. gondii* tachyzoites congenitally into the fetus leading to an anomaly in the development of the fetal brain.

From the analysis of the reports of CBC test, we observed significant alterations in the blood cell counts between T2DM patients and the healthy controls. The lower count of total WBC, neutrophils, and circulating eosinophils in diabetes patients may refer to immune dysfunction. Neutrophils are required in the course of infection for microbicidal activities with the production of Reactive Oxygen Species, lysosomal enzymes, and antimicrobial peptides. Eosinophils are the most potent immune cell during allergy and parasitic infections producing IL-4 and IL-13 cytokines. We also observed higher levels of lymphocytes and monocytes in the T2DM patients, which outlines the fact that diabetes mellitus is an inflammatory disease and leukocyte dysfunction is probable in this condition.

In our study, there was a relationship between the activation of IL-12 with *T. gondii* infection due to the significantly higher concentration of IL-12 in the infected subjects observed. This incidence may considerably be associated with the development of host immunity during the parasitic infection. IL-12 secretion is followed by the production of IFN-*γ*, which is more crucial in the Th1 mediated defense mechanism against infection. The development of host defense to *T. gondii* is greatly influenced by the activation of IL-12 potent stimulus for Th1 cell mediated immune response. *T. gondii* antigens influence the production of IL-12 from dendritic cells (DC), neutrophils, and macrophages, which activate the IFN-*γ* from NK cells and T cells. The pathway is crucial for the immunity against acute and chronic stages of toxoplasmosis. Our result also showed a lower level of IL-12 secretion in the seropositive T2DM patients than in the seropositive healthy controls although the result was not significant. This may explain an increase in the diabetes-related complications due to parasitic infection though further research is needed to address this aspect. Future studies are necessary to address the exact mechanism of immunity and cytokine signaling pathways in human toxoplasmosis.

## Figures and Tables

**Table 1:** Demographic and clinical characteristics of case group (T2DM).

Parameters		Case (T2DM) (*N* = 100)	
		Mean or *N* (range)	SD or %
Age (years)		45.72 (25–70)	11.23
Sex	Male	42	42%
	Female	58	58%
BMI (kg/m^2^)	Underweight	6	6%
	Normal	62	62%
	Overweight	20	20%
	Obese	12	12%
Fasting plasma glucose (mmol/L)		11.85	3.75
Postprandial plasma glucose (mmol/L)		19.78	5.56

## Data Availability

The datasets supporting the results and conclusion of this article were included within the manuscript file.

## Supplementary Materials

Supplementary Materials Supplementary 1. Table S1: Comparison of the complete blood count test data between different groups.

Supplementary Materials Supplementary 2. Figure S1: Correlation matrix after performing correlation analysis in the demographic and clinical data on the case group. Correlation coefficient value is labeled in red to blue color gradient. Result shows positive correlation between fasting plasma glucose (FPG) and post-prandial plasma glucose (PPG); total WBC and neutrophils. Result shows negative correlation between lymphocytes and neutrophils; neutrophils and circulating eosinophils; and total WBC and lymphocytes.

Supplementary Materials Supplementary 2. Figure S2: Correlogram matrix displaying the correlation coefficient and significance level of the correlation between different variables in the case group. The result is considered significant if *P* < 0.05.
