# Supplementary material for: Association of Chronic Toxoplasma gondii Infection with Pro-Inflamatory Cytokine Interleukin (IL)-12 Responses in Type-2 Diabetes Mellitus Patients of Bangladesh
Source: J Parasitol Res. 2023 May 8;2023:3885160. doi: 10.1155/2023/3885160 (PMC10185420; doi:10.1155/2023/3885160)
Supplement: Supplementary Materials — Supplementary 1. Table S1: Comparison of the complete blood count test data between different groups. [file 3885160.f1.pdf]

Supplementary Table 1: Comparison of the complete blood count test data between different groups

Blood parameter	Type-2 DM (N= 100)		Healthy Control (N=100)		*p*-value
	Mean	SD	Mean	SD	
**Hemoglobin (g/dL)**	13.24	1.589	11.61	1.679	<0.0001
**ESR (mm/h)**	38.78	26.54	35.40	24.53	0.4858
**Total WBC (cumm)**	8347	2046	9833	2621	0.0015
**Total RBC (million/cumm)**	5.071	0.667	4.763	0.882	0.0886
**Total Platelet (cumm)**	310960	90474	277679	66076	0.0359
**Cir. Eosinophils (cumm)**	264.9	252.0	308.1	129.9	0.0026
**Neutrophils (%)**	63.86	9.108	68.65	12.01	0.0128
**Lymphocytes (%)**	29.50	8.320	25.29	11.45	0.0204
**Monocytes (%)**	3.380	1.462	2.809	1.109	0.0067
**Eosinophils (%)**	3.220	2.773	3.353	1.647	0.0444
**Basophils (%)**	0.040	0.1969	0.0196	0.140	0.6630
**MCV (fL)**	78.43	7.767	79.78	9.901	0.1146
**RDW-SD (fL)**	38.00	3.775	38.82	6.877	0.0214
**HCT (%)**	39.53	4.751	36.86	5.680	0.0012
**MPV (fL)**	10.72	2.521	9.400	1.757	<0.0001
**PDW (fL)**	13.09	3.921	14.78	2.498	<0.0001
