# Supplementary figures and images for: Association of Chronic Toxoplasma gondii Infection with Pro-Inflamatory Cytokine Interleukin (IL)-12 Responses in Type-2 Diabetes Mellitus Patients of Bangladesh
Source: J Parasitol Res. 2023 May 8;2023:3885160. doi: 10.1155/2023/3885160 (PMC10185420; doi:10.1155/2023/3885160)

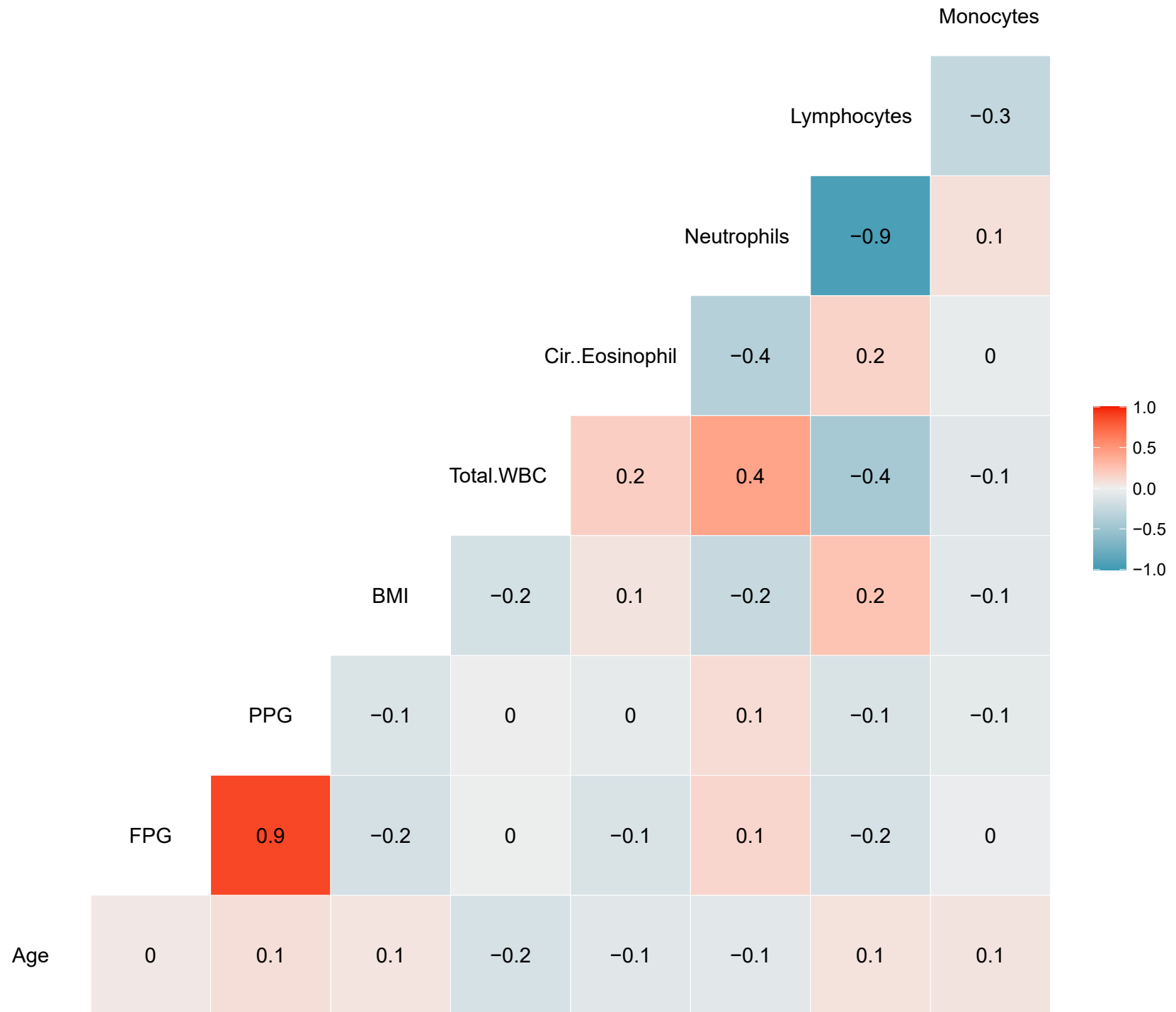

Supplement: Supplementary Materials — Supplementary 2. Figure S1: Correlation matrix after performing correlation analysis in the demographic and clinical data on the case group. Correlation coefficient value is labeled in red to blue color gradient. Result shows positive correlation between fasting plasma glucose (FPG) and post-prandial plasma glucose (PPG); total WBC and neutrophils. Result shows negative correlation between lymphocytes and neutrophils; neutrophils and circulating eosinophils; and total WBC and lymphocytes. [file 3885160.f2.pdf]

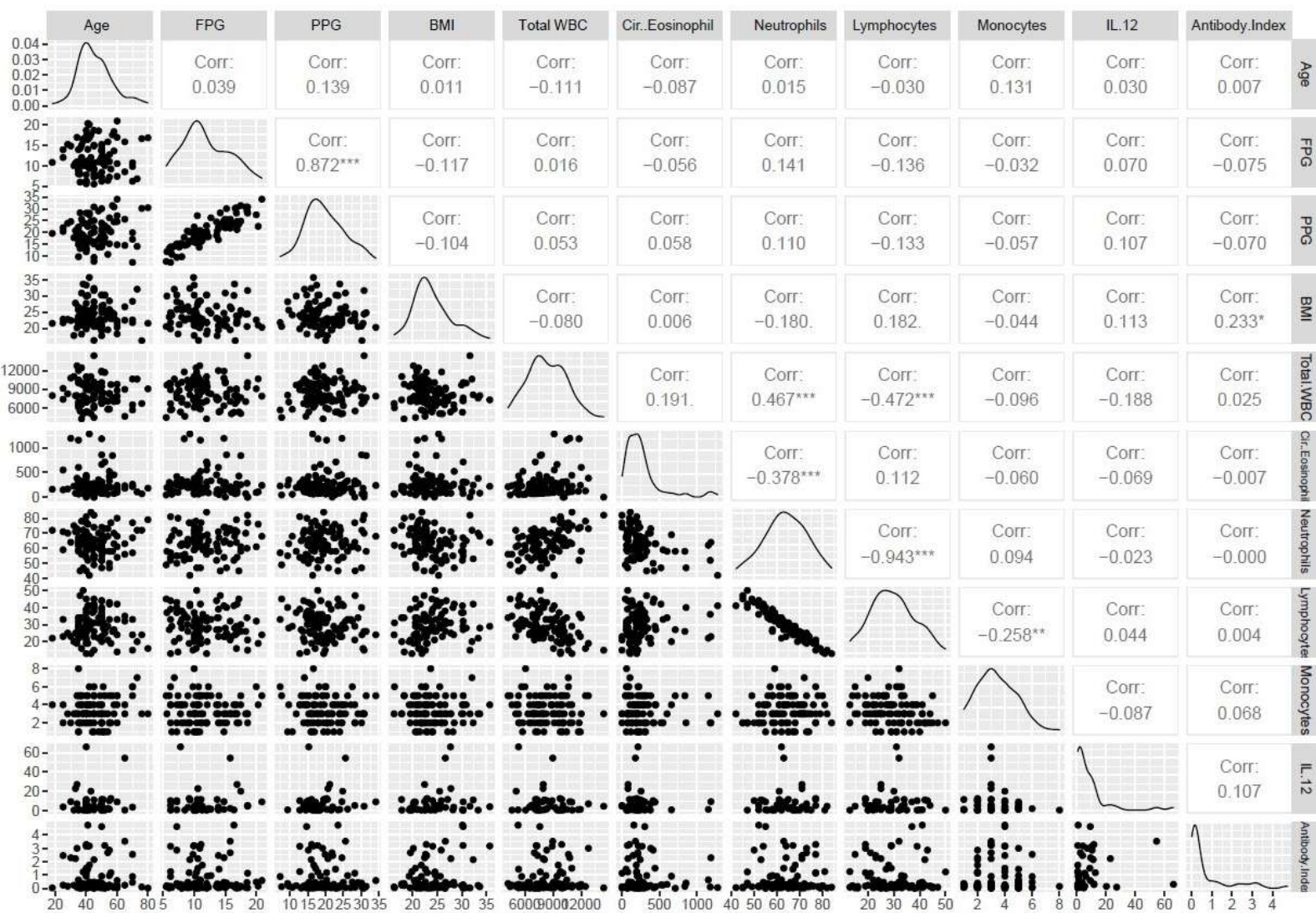

Supplement: Supplementary Materials — Supplementary 2. Figure S2: Correlogram matrix displaying the correlation coefficient and significance level of the correlation between different variables in the case group. The result is considered significant if P < 0.05. [file 3885160.f3.pdf]
